# ASSESSING THE INTEGRATION OF AGE-FRIENDLY DESIGN RESEARCH INTO THE DESIGN OF HEALTH CARE ENVIRONMENTS

**DOI:** 10.1093/geroni/igae098.2023

**Published:** 2024-12-31

**Authors:** Margaret Calkins

**Affiliations:** IDEAS Institute, Cleveland Heights, Ohio, United States

## Abstract

Older adults account for roughly 2/3rds of all patient days in hospitals, and more than 90% of days in post-acute care settings. It is not just the care delivered by clinicians that determines outcomes. Different environments – organizational, social, as well as physical – also have significant impacts on a variety of health and well-being outcomes. Great strides have been made over the past two decades in the design of residential and care environments for older adults, and in the research that evaluates these designs. Research has examined these environments at many scales from overall design concepts (for example, the medical model versus residential design approaches) down to details such as the design of supportive handrails and hardware. This presentation will give a brief overview of this research and explore SAGE (Society for the Advancement of Gerontological Environments) Federation’s post-occupancy process for evaluating residential and care environments for older adults, which has been conducted annually for the past 20 years. However, much less focus has been paid to making other healthcare environments, particularly acute care hospitals, more age-friendly. The Island Health system in Vancouver, Canada, set out to change this and designed the Royal Jubilee Hospital to be age-friendly from pre-admission through discharge. The presentation will also explore the process that Island Health used for decision-making, and how this has influenced subsequent new construction.

